# Evolution of a Higher Intracellular Oxidizing Environment in *Caenorhabditis elegans* under Relaxed Selection

**DOI:** 10.1371/journal.pone.0065604

**Published:** 2013-06-11

**Authors:** Joanna Joyner-Matos, Kiley A. Hicks, Dustin Cousins, Michelle Keller, Dee R. Denver, Charles F. Baer, Suzanne Estes

**Affiliations:** 1 Department of Biology, Eastern Washington University, Cheney, Washington, United States of America; 2 Biology Department, Portland State University, Portland, Oregon, United States of America; 3 Department of Zoology and Center for Genome Research and Biocomputing, Oregon State University, Corvallis, Oregon, United States of America; 4 Department of Biology and University of Florida Genetics Institute, University of Florida, Gainesville, Florida, United States of America; Fred Hutchinson Cancer Research Center, United States of America

## Abstract

We explored the relationship between relaxed selection, oxidative stress, and spontaneous mutation in a set of mutation-accumulation (MA) lines of the nematode *Caenorhabditis elegans* and in their common ancestor. We measured steady-state levels of free radicals and oxidatively damaged guanosine nucleosides in the somatic tissues of five MA lines for which nuclear genome base substitution and GC-TA transversion frequencies are known. The two markers of oxidative stress are highly correlated and are elevated in the MA lines relative to the ancestor; point estimates of the per-generation rate of mutational decay (ΔM) of these measures of oxidative stress are similar to those reported for fitness-related traits. Conversely, there is no significant relationship between either marker of oxidative stress and the per-generation frequencies of base substitution or GC-TA transversion. Although these results provide no direct evidence for a causative relationship between oxidative damage and base substitution mutations, to the extent that oxidative damage may be weakly mutagenic in the germline, the case for condition-dependent mutation is advanced.

## Introduction

It is well known that the genomic mutation rate and spectrum vary within and among taxonomic groups [1], [2], but the relative influences of exogenous (environmental) versus endogenous (genomic or physiological) factors are poorly understood, particularly in multicellular eukaryotes. Among endogenous factors, one proposed cause of mutation is the oxidative damage to DNA that can result from an imbalance between free radical production and detoxification/repair ( = oxidative stress). Oxidative damage to DNA takes many forms, including base modification, strand breaks, large genome rearrangements, large deletions, crosslinks, and protein-DNA adducts [3], [4], [5], [6], [7], [8]. The best studied form of damage-induced mutation results from the oxidation of guanosine, which creates a readily-identified product (8-oxo-7,8-dihydro-2′-deoxyguanosine, or 8-oxodG) that can cause G:C-T:A transversions if not removed by repair enzymes [9], [10], [11], [12]. Oxidized guanine bases have been termed the “hallmark of oxidative stress” given the high incidence of G-T transversions in aging or cancerous somatic tissues [13]. There are repair mechanisms for most forms of oxidative DNA damage, including 8-oxodG [3], [4], [5], [6], [7], [8], yet oxidative damage is known to contribute to aging and a multitude of diseases [14], [15] and to mutation accumulation in somatic tissues [13], [16], [17]. The causative relationship between oxidative stress and heritable mutation, however, is more tenuous [18].

Here we explore the relationship between natural selection, spontaneous mutation, and two important markers of oxidative stress, 8-oxodG and steady-state free radical level, in a set of five “mutation-accumulation” (MA) lines of the rhabditid nematode *Caenorhabditis elegans* for which the nuclear genome base substitution and G:C-T:A transversion frequencies are known [19] relative to a common ancestor. Heritable mtDNA mutations have not been characterized in this set of MA lines. The principle of a mutation accumulation experiment is simple: natural selection is inefficient when effective population size is small, so mutations with effects on fitness less than about 1/4N_e_ will accumulate at the neutral rate [20], [21], [22]. Populations experiencing relaxed selection will, on average, evolve toward lower fitness. Thus, if the mean of a trait changes with MA, the simplest explanation is that deleterious mutations are moving the trait away from its selective optimum.

We address two proximate questions. First, what is the cumulative effect of hundreds of generations of evolution in the near absence of natural selection on the level of oxidative stress experienced by an individual worm, and second, is there a detectable relationship between the relative degree of oxidative stress experienced by an average individual of a given MA line and the spectrum of base substitution mutations present in that line? These proximate questions are motivated by two deeper, unresolved issues in evolutionary biology. First, although variability in oxidative damage is often invoked as an important underlying cause of variation in the rate and spectrum of molecular evolution [23], [24], the connection between variability in oxidative damage and variability in heritable mutation is weak. Second, evidence is accumulating that, at least in some cases, the genomes of individuals in poor physiological condition tend to mutate more readily than do genomes of individuals in good condition [25], [26], [27]. One cause of poor condition is a pre-existing load of deleterious mutations. If it can be established that (1) conditions that reduce fitness lead to an increase in oxidative stress and (2) an increase in oxidative stress leads to an increase in the rate and/or a change in the spectrum of heritable mutations, then these hypotheses will be tied together and independently strengthened.

We found that nematodes from MA lines exhibited higher levels of steady-state oxidative stress in the soma than did nematodes from the ancestral control. Conversely, the correlation between the measures of oxidative stress and the frequencies of base substitution or G-to-T transversions in the nuclear genome was small and not significantly different from zero.

## Materials and Methods

### (i) Experimental Lines

We studied five *C. elegans* MA lines and their common ancestor (MA generation 0, or “G0”) that were generated as part of a large MA experiment [28]. These five particular lines were chosen because whole-genome sequence data are available [19], [29]; the nuclear base substitution rates for these MA lines indicated more G:C-T:A transversions than observed in nature, a pattern that could be interpreted as evidence of elevated oxidative stress in the MA lines [19], particularly since *C. elegans* may have limited DNA repair capabilities compared to other metazoans [30], [31]. The MA lines are derived from a single, highly inbred N2 strain hermaphrodite; the lines independently experienced 250 generations of serial transfer (a bottleneck; 250 MA generations) of a single individual [32]. Under these conditions, the effective population size, N_e_≈1 and selection is minimally efficient. Since mutations with selective effect, *s*<1/4N_e_ are effectively neutral [20], [21], all but the most highly deleterious mutations (s>0.25) are expected to accumulate at the neutral rate. Details of the MA protocol and the mutational declines in fitness in the MA lines (at G200) relative to the “unmutated” ancestor (G0) are reported in [28].

### (ii) Measurement of Steady-state Reactive Oxygen Species (ROS) Levels by Confocal Microscopy

Cryogenically preserved stocks of the MA lines (G250) and common ancestor (G0) were thawed and nematodes were allowed to recover for one week under standard laboratory conditions at 20°C, with regular population transfers to fresh 15 mm nematode growth medium (NGM) Petri plates inoculated with OP50-1 *Escherichia coli*. Two independent, age-synchronous populations of each line were generated by bleaching [33]; half of each population was reserved to create line-specific internal control groups. We performed confocal image analysis on live young adult nematodes using our previously described methods [34], [35], [36]. Briefly, nematodes were incubated for 24 hours at 20°C in the presence or absence of 10 µM MitoSOX Red (in water; Molecular Probes Inc.), a mitochondria-targeted dye that fluoresces when in contact with (total) mitochondrial oxidants, reflecting both ROS generation and ROS scavenging [37]. Total oxidant production was measured in the pharyngeal bulb, a tissue that is particularly suited for assessment of oxidative stress because it has high metabolic activity and dense populations of mitochondria [38], the primary source of endogenous ROS. It is important to note that the ROS data described mitochondrial oxidative stress while the mutation data were derived from the nuclear genome. Although mitochondrial ROS can damage cytoplasmic and nuclear components [39], the relationship between mitochondrial function and nuclear genetic damage is not straightforward, owing to variation in the stability, longevity and diffusion properties of different ROS [40] and because low levels of ROS may alter DNA repair activity [41], [42], [43].

For each line, fluorescent z-stack images of the pharyngeal bulbs of 15-20 treatment (+MitoSOX) and 5 control (-MitoSOX) nematodes that had been immobilized by levamisole were captured at 60X magnification using a high resolution wide field Core DV system (Applied Precision™; Oregon Health and Sciences University Advanced Light Microscopy Core Facility, Portland, OR). Deconvolution-optimized images were used to quantify relative ROS levels by manually enclosing the terminal pharyngeal bulb within each image and obtaining the maximum intensity of the area using ImageJ software (National Institutes of Health). Final ROS levels for each line were calculated as the difference between pharyngeal bulb intensity in labeled and unlabeled control worms from each line.

### (iii) Measurement of 8-oxodG Levels by Enzyme-Linked ImmunoSorbent Assay (ELISA)

We measured oxidative damage in 12-day-old nematodes because oxidative damage in general is more reliably detected in older nematodes [44], [45] and significant differences in steady-state 8-oxodG content have been harder to detect in young adult [39] and mixed-stage nematodes [46]. Additionally, our preliminary work with a mutant strain (the *mev-1* mutant strain) that has constitutively elevated oxidative stress [47], [48] indicated that while steady-state ROS levels were significantly elevated in *mev-1* compared to N2 individuals in the young adult stage (S.E., unpubl. results), differences in 8-oxodG were not detected in young adult nematodes (J.J.-M., unpubl. results). Frozen stocks for the MA lines and G0 ancestor were thawed; five individuals from each line were randomly selected to initiate biological replicates, which were carried through three generations of single-individual descent in standard conditions and then expanded to a large population and age-synchronized [33]. Upon reaching the L4 larval stage, each population (five populations for each MA line and the G0 ancestor) was transferred to NGM plates containing 40 µM 5-fluoro-2′-deoxyuridine (FUdR) to prevent mixing of the focal population with its progeny. FUdR inhibits DNA and RNA synthesis; since most of the cells in an adult nematode are postmitotic, treatment with FUdR inhibits the production of viable progeny and is routinely used in studies of wild-type and mutant nematode strains [38], [49], [50], [51], [52]. While FUdR treatment does affect an assortment of metabolic processes in *C. elegans* [53] and likely alters mitochondrial DNA replication and mitochondrial biogenesis, it is not clear whether FUdR treatment can be expected to alter oxidative stress since it did not alter mitochondrial morphology [54] or antioxidant enzyme expression [55] in nematodes. All nematodes were maintained on FUdR-containing plates until they were 12 days old, at which point the nematodes were washed in M9 buffer, flash-frozen and stored at −80°C.

To minimize DNA oxidation during sample preparation [56], we used the chaotropic sodium iodide method [57] with a DNA Extractor TIS kit (Wako) with lengthened ethanol incubation and RNase steps. We quantified DNA and confirmed the absence of RNA using a Qubit Fluorometer (Life Technologies); one sample that had detectable RNA levels was discarded. Samples and a standard curve of an oligonucleotide containing 8-oxodG (Trevigen) were diluted in a TE buffer (with the Wako oxidation inhibitor) and incubated with intermittent vortexing for 10 minutes with an equal volume of Reacti-Bind (Pierce DNA coating solution, Thermo Scientific).

We conducted an “indirect” ELISA, plating the samples in triplicate (100 µL per well) in MaxiSorp 96-well plates (Nunc) and incubating the plates overnight at room temperature on an orbital shaker (Reacti-Bind facilitates the binding of oligonucleotides to the 96-well plates). The next day, wells were washed with phosphate buffered saline with 0.05% Tween-20. Wells were then subjected to three sequential incubation steps at 37°C with shaking, with multiple washes between each step: 1) one hour in blocking solution (0.5% fetal calf serum), 2) two hours with the anti-8-oxodG primary antibody (mouse monoclonal antibody, Clone 2E2, Trevigen), and 3) two hours with a secondary antibody (goat anti-mouse IgG, alkaline phosphatase conjugated, Sigma). Wells were incubated in the dark (room temperature) with p-Nitrophenylphosphate Alkaline Phosphatase Substrate solution (generates yellow color when it reacts with the alkaline phosphatase conjugated to the secondary antibody; Vector Laboratories); absorbance was measured every 30 minutes at 405 nm wavelength (Molecular Devices). The signal increased in intensity for 2.5 hours until reaching a plateau. Data from the 2.5 hour read were corrected by subtracting from each data point the average optical density of three blank wells (TE buffer) in each plate. The standard curves were modeled by the one-site saturation, ligand-binding curve fit in SigmaPlot 11 (Systat Software, Inc.); we calculated the nanograms of DNA equivalents per well and then used the copy number template from the URI Genomics and Sequencing Center (http://www.uri.edu/research/gsc/resources/cndna.html) to calculate the number of damaged bases per well. Data are reported as ×10^9^ damaged bases per nanogram of DNA.

### (iv) Mutation Rate Estimates and Data Analysis

Per-generation frequencies of base substitutions (µ_BS_) were calculated for each line by dividing the number of new base substitutions that arose during MA by the product of the total number of nucleotides sequenced and the number of generations of MA [19]. Per generation frequencies of G-to-T transversions (µ_G-to-T_) were calculated by dividing the number of G-to-T mutations by the product of the number of G C sites considered and the estimated number of generations experienced by each MA line [19].

We compared the traits (ROS and 8-oxodG levels) between the MA lines and the G0 ancestor using restricted maximum likelihood (REML) with the MIXED procedure of SAS (v. 9.3). The independent variable MA treatment (MA versus G0 ancestor) is a fixed effect while Line is a random effect. We analyzed the model *Trait = MA Treatment*+*Line(MA Treatment).* To test whether ROS and 8-oxodG levels in each MA line differed from the levels in the G0 ancestor we constructed contrasts using the model *Trait = Line*. We calculated Spearman’s rank correlation coefficients to evaluate the relationships between ROS levels and 8-oxodG content, and between these traits and the frequencies of nuclear base-substitutions and G-to-T transversions (JMP 9, SAS Institute). Correlation analyses were conducted using line means for each trait.

To calculate the per-generation rate of change of the trait, ΔM, we divided each data point by the G0 trait mean and estimated the slope of the relationship between trait value and generation using the linear model *Trait = Generation+Line(MA Treatment)+error*. The among-line variance was calculated separately for each MA treatment group and constrained to equal zero in the G0. We compared a model in which the within-line (error) variance was allowed to vary between MA treatment groups against a model with a single within-line variance by likelihood-ratio test (LRT), in which twice the difference in log-likelihoods of the two models is asymptotically chi-square distributed with degrees of freedom equal to the difference in the number of parameters estimated in the two models ( = 1 df). If the LRT was not significant (*p*>0.05), we report results from the model with a single error variance; otherwise we report results from the model with separate within-line variances in the two MA treatments.

## Results

**Figure 1:**
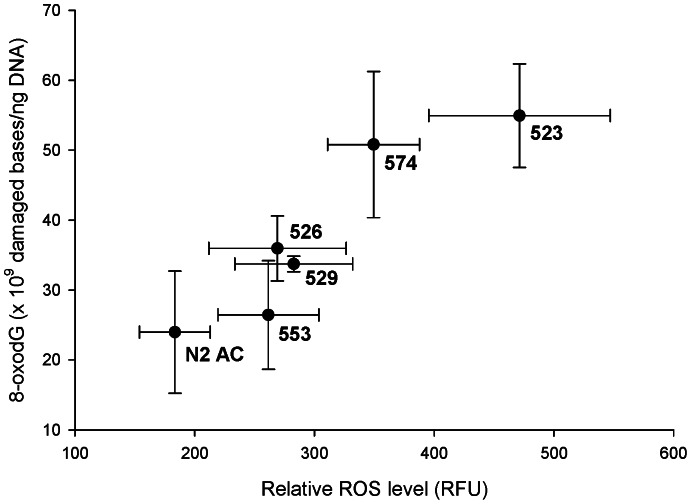
Bivariate relationship of line means for net *in vivo* ROS level and 8-oxodG content. Relative reactive oxygen species (ROS) levels are reported in relative fluorescence units (RFU); quantity of 8-oxo-7,8-dihydro-2′-deoxyguanosine, or 8-oxodG, are reported as ×10^9^ damaged bases per nanogram of DNA. Line means of the two metrics were significantly correlated (Spearman’s ρ = 0.943, *p* = 0.017 with all lines present). Bars represent one standard error. “N2 AC” is the N2 ancestor (progenitor of MA lines, Generation 0); remaining data labels are the Baer MA line numbers.

Averaged over all lines, the MA lines had significantly higher *in vivo* ROS levels compared to the G0 ancestor (*F* = 4.99, *p* = 0.0342; Table 1), with ΔM = 0.0032 (0.0011)/generation, mean (SEM) (ΔM different from zero, *p*<0.02). Two of the MA lines had individually significantly higher levels of steady-state ROS than did the G0 ancestor (line 523, *p*<0.0001; line 574, *p* = 0.0241). The MA lines also had marginally higher mean 8-oxodG content than did the G0 ancestor (*F* = 3.03, *p* = 0.0964; Table 1), with ΔM = 0.0028 (0.0016)/generation (0.09<*p*<0.1). The difference in 8-oxodG level was individually significant for line 523 (*p* = 0.0088 versus G0 ancestor) and line 574 (*p* = 0.0207). Line means of ROS levels and 8-oxodG content were strongly positively correlated (including the G0: Spearman’s ρ = 0.943 (95% confidence interval (CI): 0.560, 0.993), p = 0.017; only the MA lines: ρ = 0.90, p = 0.083 (95% CI: 0.086, 0.993); Figure 1).

**Table 1:** Estimates of oxidative stress and mutation frequency.

Line†	Relative ROS (SE)‡	8-oxodG (SE)§	µ_BS_ ¶	µ_G-TO-T_
523	471.4 (75.8)*	54.92 (7.4)*	3.163E-09	3.987E-05
526	269.2 (57.3)	35.95 (4.6)	2.446E-09	1.417E-05
529	282.8 (49.2)	33.72 (1.1)	1.845E-09	2.080E-05
553	261.5 (48.6)	26.43 (7.8)	2.890E-09	3.687E-05
574	350.4 (38.4)*	50.79 (10.4)*	1.757E-09	1.982E-05
MA mean	328.2 (39.6)	40.72 (5.4)	–	–
N2 ancestor	192.9 (29.6)	23.97 (8.7)	–	–

† Baer mutation accumulation (MA) line number from the Baer et al. (2005) experiment [28].

‡ Relative reactive oxygen species (ROS) levels expressed as means (standard error) of relative fluorescence units.

* Indicates significantly different from N2 ancestor.

§ Means (standard error) of 8-oxo-7,8-dihydro-2′-deoxyguanosine, or 8-oxodG, are reported as ×10^9^ damaged bases per nanogram of DNA.

¶ See Materials and Methods for calculations of point estimates of the frequencies of base substitutions (µ_BS_) and G-to-T transversions (µ_G-TO-T_).

Estimates of base substitution frequencies in the five MA lines are reported in Table 1. Steady-state ROS level was not correlated with the frequency of base substitutions (µ_BS_; Spearman’s ρ = 0.000 (95% CI: −0.882, 0.882), p = 1.0) nor to the frequency of G-to-T transversions (µ_G-to-T_; Spearman’s ρ = 0.300 (95% CI: −0.792, 0.935), p = 0.683). Similarly, 8-oxodG level was not correlated with either mutational measure (µ_BS_: Spearman’s ρ = 0.100 (95% CI: −0.858, 0.903), p = 0.95; µ_G-to-T_: Spearman’s ρ = 0.100 (95% CI: −0.858, 0.903), p = 0.95).

## Discussion

This study was undertaken with two proximate goals in mind: first, to understand the cumulative effects of minimally efficient selection on the intracellular oxidative environment, and, second, to investigate the relationship between variation in the oxidative environment and variation in the rate and/or spectrum of base substitution mutations in the nuclear genome. Denver et al. [19] sequenced the nuclear genomes of seven MA lines derived from the N2 ancestor, but we were unable to obtain sufficient material from two of those lines to do the assays reported here. Obviously, with only five MA lines the statistical power is low and for an effect to be detectable it must be correspondingly large.

In fact, the effect of the MA environment on total ROS level is sufficiently large to produce a statistically significant result: the imbalance between free radical production and detoxification/repair increased significantly over 250 generations of relaxed selection (ROS: ΔM = 0.0032/generation, p<0.02). The effect of the MA environment on 8-oxodG is nearly sufficiently large (0.0028/generation; p<0.1) and has the same magnitude of the per-generation change as ROS level, consistent with the very high correlation of line means between the two variables (Spearman's ρ>0.9).

The small number of lines raises the concern that any statistically significant result is a false positive. Although that possibility cannot be ruled out, we note that two of the MA lines we attempted to assay had such low fitness that we were unable to conduct the assays. Because the lines we were able to assay are upwardly biased relative to a random sample of MA line fitnesses and because increased susceptibility to oxidative stress is commonly associated with low fitness [58], [59], there is at least some reason to believe that the results of these assays are likely to be conservative compared to the results of an assay of MA lines randomly sampled with respect to fitness.

In contrast to the strong association of the two measures of oxidative stress with the MA environment, there is no strong association between either oxidative stress measure and the total frequency of base substitutions (µ_BS_) or G:C to T:A transversions (µ_G-to-T_). Although the results of this study and results from other studies involving *C. elegans* [60], [61] and *Drosophila* [62] clearly show that strong and significant results can be detected with a small number of MA lines, there are several reasons why the failure to detect a strong relationship between oxidative stress and the frequency of base substitutions should not be surprising. First, the measures of oxidative stress reported here were measured at the endpoint of 250 generations of evolution under relaxed selection, whereas mutations accumulated in the genome over the entire 250 generations. For example, it is possible that the two lines with individually significant increases in ROS level experienced mutations that affected some feature of ROS metabolism only recently, in which case the increased ROS would have had little time to contribute to the mutational process. Second, since mtDNA mutations are not characterized in these lines, we cannot assess the potential contribution of mitochondrial oxidative stress to mutational processes in mtDNA. Third, the (nuclear) mutation rate (which is distinct from the frequency of mutations) does not differ significantly between MA lines; the differences among lines in base substitution frequency is no more extreme than expected if mutations are Poisson distributed among lines with a uniform mutation rate [19]. The fact that mutation rate does not differ between lines suggests that there is no variable process underlying the base substitution process. Fourth, oxidative damage is only one contributor to the base substitution mutation process; base misincorporation resulting from polymerase errors also contributes. Our marker of oxidative damage, 8-oxodG in total DNA pools (nuclear and mitochondrial), is only one potential cause of transversion mutations and thus may be a less reliable indicator of mutation resulting from oxidative damage than previously thought [13]. Finally, it is important to note that we only considered base substitutions in the nuclear genome, and that there is evidence that the mutagenic effects of oxidative stress primarily result in other types of mutations in somatic tissues, including large deletions and genome rearrangements [63], [64].

An additional consideration is that we measured oxidative damage in the soma, whereas we measured heritable mutations that occurred in the germline. Elements of the DNA repair process [1], [65], [66], [67], [68], [69], [70], [71] and antioxidant defense systems [18] are known to differ between the soma and the germline; however, evidence is emerging that somatic oxidative stress is associated with and may even contribute to DNA damage and/or mutation in the germline [18]. However, to the extent that the estimates of ΔM of ROS and 8-oxodG reported here are trustworthy, there is every reason to expect that the processes responsible for maintaining the oxidative millieu of the germline will have undergone similar mutational degradation over the 250 generations of relaxed selection. If germline oxidative metabolism has not undergone similar mutational degradation, it could only be for one of two (nonexclusive) reasons: either the mutational target presented by the germline is for some reason much smaller than the target presented by the soma, in which case the inevitable mutational decay would take longer, or the fraction of mutations that can affect germline oxidative metabolism and are strongly deleterious (4N_e_s<1) is much larger. The not-unreasonable possibility that mutations affecting germline oxidative metabolism are extremely deleterious has an important implication: it argues against variation in oxidative metabolism having an important role in the process of molecular evolution.

The study reported here was ultimately motivated by the possibility that oxidative stress is a causal factor underlying condition-dependent mutation. The results provide no direct support for such a causal relationship, at least not with respect to base substitutions in the nuclear genome. However, to the extent that oxidative stress may be weakly mutagenic and this study simply lacked sufficient power to detect the relationship, the apparently rapid mutational degradation of the mechanism underlying control of cellular oxidative processes provides some succor for the hypothesis that the mutational process is condition-dependent.
